# Highly Immunoreactive IgG Antibodies Directed against a Set of Twenty Human Proteins in the Sera of Patients with Amyotrophic Lateral Sclerosis Identified by Protein Array

**DOI:** 10.1371/journal.pone.0089596

**Published:** 2014-02-26

**Authors:** Caroline May, Eckhard Nordhoff, Swaantje Casjens, Michael Turewicz, Martin Eisenacher, Ralf Gold, Thomas Brüning, Beate Pesch, Christian Stephan, Dirk Woitalla, Botond Penke, Tamás Janáky, Dezső Virók, László Siklós, Jozsef I. Engelhardt, Helmut E. Meyer

**Affiliations:** 1 Department of Medical Proteomics/Bioanalytics, Medizinisches Proteom-Center, Ruhr-University Bochum, Bochum, Germany; 2 Institute for Prevention and Occupational Medicine of the German Social Accident Insurance, Institute of Ruhr-University Bochum, Bochum, Germany; 3 St. Josef-Hospital, Ruhr-University Bochum, Bochum, Germany; 4 Department of Medical Chemistry, University of Szeged, Szeged, Hungary; 5 Institute of Clinical Microbiology, University of Szeged, Szeged, Hungary; 6 Institute of Biophysics, Biological Research Center of the Hungarian Academy of Sciences, Szeged, Hungary; 7 Department of Neurology, University of Szeged, Szeged, Hungary; 8 Leibniz-Institut für Analytische Wissenschaften - ISAS - e.V., Dortmund, Germany; University of Jaén, Spain

## Abstract

Amyotrophic lateral sclerosis (ALS), the most common adult-onset motor neuron disorder, is characterized by the progressive and selective loss of upper and lower motor neurons. Diagnosis of this disorder is based on clinical assessment, and the average survival time is less than 3 years. Injections of IgG from ALS patients into mice are known to specifically mark motor neurons. Moreover, IgG has been found in upper and lower motor neurons in ALS patients. These results led us to perform a case-control study using human protein microarrays to identify the antibody profiles of serum samples from 20 ALS patients and 20 healthy controls. We demonstrated high levels of 20 IgG antibodies that distinguished the patients from the controls. These findings suggest that a panel of antibodies may serve as a potential diagnostic biomarker for ALS.

## Introduction

Amyotrophic lateral sclerosis (ALS), also known as Lou Gehrig’s disease, is a progressive neurodegenerative disorder characterized by the loss of lower motor neurons in the brain stem and spinal cord and upper motor neurons in the motor cortex [1]. ALS is mainly a degenerative disorder of the motor system, although this condition can also be accompanied by cognitive impairment. ALS is generally a sporadic disease (SALS), but a genetic component with an autosomal dominant inheritance has been found in 5–10% of ALS patients [2]. In contrast to the advances made in the genetic epidemiology of ALS, less is known about potential environmental factors and their interaction with genetic susceptibility factors [2], [3].

Diagnostic criteria have been developed to improve ALS disease classification. However, early classification is not likely to be reliable, and most patients have a delay of nearly 1 year from the occurrence of the first symptoms until diagnosis. Thus, additional diagnostic tools are needed to detect ALS at earlier time points. Molecular markers may support this effort if these markers can segregate ALS patients from non-diseased subjects using minimally invasive methods.

A number of studies on ALS have been carried out using post-mortem tissues, although the target organ of ALS is not accessible for the early detection of this disease. However, the serum may serve as a proxy tissue to detect diagnostic biomarkers, and recent techniques offer the possibility of detecting genomic, proteomic, or other changes in the blood during disease progression, thereby providing new insight into the pathological pathways of ALS [4]. The humoral immune response is increasingly a focus of ALS research, and data suggest that multiple antibodies directed against different motor neuron structures may play some role in the motor neuron degeneration seen in ALS [5], [6]. Furthermore, ALS patients have been shown to mount a humoral immune response that is harmful to motor neurons. For example, the injection of IgG from SALS patients into mice revealed the specific labeling of murine motor neurons [5], [6].

To identify potential autoantibodies, earlier studies have pursued hypothesis-driven approaches. In one such approach, putative candidate autoantigens were coated onto an ELISA plate and incubated with patient serum samples [4], [7]–[14]. In contrast to such ELISAs, we set out to utilize protein microarrays, which offer the possibility of the simultaneous analysis of 9,480 putative autoantigens with the additional advantages of homogeneous technical conditions and lower costs per antigen.

In the present study, we employed protein microarrays to evaluate serum samples from 20 ALS patients and 20 non-diseased controls, and the antibody-binding reactions were studied in order to identify antibodies that may distinguish ALS cases from controls.

## Materials and Methods

### Ethical Statement

The study was approved by the ethics committee at the Ruhr-University Bochum, Germany and the ethics committee at the Medical Faculty at the University of Szeged, Hungary. All participants provided written, fully informed consent to participate in this study. The relevant documents relating to this process are filed at the Department of Neurology, University of Szeged, Hungary. Only the anonymous data and materials from the Hungarian participants (patients with ALS and controls) were provided to the scientists carrying out the research. The data concerning this study were stored separately from the hospital charts of the patients.

### Subjects and Samples

This ALS study had a cross-sectional design including 20 ALS cases and 20 controls. All subjects were recruited at the Department of Neurology, University of Szeged, Hungary. Patients were not eligible for the study if they demonstrated cognitive impairment (Mini-Mental State Examination score <27), any drug addiction, or were HIV-positive. All participants had to be able to understand and speak the Hungarian language fluently. ALS cases were eligible if their diagnosis was validated according to the El Escorial revised diagnostic criteria for the diagnosis of ALS [15]. The controls were disease-free subjects (volunteer blood donors or other healthy volunteers) who were unrelated to the ALS cases and who were matched to the ALS cases by age and gender. A neurologist enrolled and diagnosed eligible subjects according to a standardized assessment protocol, including a neurological examination, socio-demographic questions, and disease-related questionnaires. For all ALS cases, the severity of the disease was assessed using a revised ALS functional rating scale (ALSFRS) and modified manual muscle testing [16]. All of the ALS cases exhibited SALS.

### Blood Collection and Routine Laboratory Analysis

Venous blood samples were obtained in Hungary from an antecubital vein through an indwelling catheter for the protein microarray experiments and for the determination of routine laboratory parameters. Blood samples for routine laboratory parameters were collected in EDTA tubes containing 100 µL of 0.5% sodium disulfide solution and in 5 mL tubes for serum preparation (Kabevette® V serum Gel S831 V, Kabe Laborstechnik GmbH, Nümbrecht-Elsenroth, Germany). Routine clinical variables were determined using established routine protocols. For protein microarray experiments, serum was prepared in accordance with the manufacturer’s protocol (Kabe Laborstechnik GmbH). The sera were stored in small aliquots at −80°C until transport and analysis in Germany.

### Protein Microarray

Before performing the protein microarray, the slides (ProtoArray, Life Technologies, Carlsbad, USA) were equilibrated at 4°C for 15 min and then for an additional 15 min at room temperature. Only 10 µL of serum was used for each protein microarray experiment. Blocking, serum incubation, and washing steps were carried out as described in the manufacturer’s protocol (Life Technologies), with the exception that an automatic slide washer (M2-Automation, Berlin, Germany) was used. Image acquisition of the processed protein microarrays was performed with an Array-Scanner FR202 using CCD Technology (Strix Diagnostics GmbH, Berlin, Germany).

### Raw Data Acquisition

The raw fluorescence intensity data were acquired using StrixAluco 3 microarray image analysis software (Strix Diagnostics GmbH, Berlin, Germany). For this purpose, the lot-specific GAL file (GenePix® Array List file, obtained from Life Technologies’ ProtoArray web site http://www.lifetechnologies.com) was imported into the software, and all microarray image files were processed automatically in batch format using the StrixAluco batch mode. This resulted in a set of 40 GPR files (GenePix® Results, 1 file for each microarray) containing all of the raw intensity data.

### Selection of Highly Immunoreactive IgG Antibodies Directed against Human Proteins

The method used in this study was an adaption of the approach proposed by Jiang *et al.* [17]. For statistical analysis, the acquired raw data were imported into R (http://www.r-project.org/, [18]) via the Bioconductor (http://www.bioconductor.org/, [19]) package limma [20]. After joint preprocessing (the preprocessed data are available at www.medizinisches-proteom-center.de/May_et_al), approximately one-third of the microarrays (6 ALS vs. 6 NDCs) were randomly selected as the test set, and the remainder (14 ALS vs. 14 NDCs) were used as the training set. The following selection procedure (“feature selection”) was applied to the training set only: for each protein feature, a “minimum M-Statistic” p value (“M Score” [21]–[23]) was computed. All of the protein features were then sorted by means of their M Score values in order to pre-select the 300 proteins with the lowest (i.e., best) M Scores (corresponding average M Score cut-off: 0.004566). The set of proteins resulting from this univariate preselection was narrowed down by multivariate selection using a random forest (RF) classifier wrapped with a backward elimination approach (“gene shaving” (GS), [17], [24], [25]). Subsequently, the selected features were verified using the test set for classification accuracy estimation. The whole procedure, including the training/test set sampling, univariate/multivariate selection, and verification, was repeated 100 times (100 “subruns”). The 20 most frequently obtained features were then finally selected (“frequency of selections”-based approach [26]). As verification of this candidate biomarker panel, 100 RF classifications using 100 redrawn training and test set splits were performed. Additionally, the final selected set of highly immunoreactive antibodies was re-verified using an alternative data analysis approach (Prediction Analysis of Microarrays, PAM [27], [28]); refer to the Supporting Information for a detailed description of these methods.

## Results

### Characteristics of the Study Groups

Details about the study groups are provided in Table 1. The median age of the cases and controls was 60 years, 50% were female, and 55% of both groups had never smoked. The average ALSFRS was 39 (range 16–46). The median time since ALS diagnosis in the ALS cases was 18 months (range 2–60). None of the ALS patients exhibited severe signs of cognitive impairment (median MMS 29.5).

**Table 1:** Characteristics of the study population (n = 40).

		Total		ALS patients		Non-diseased controls	
		N	%/Median (IQR^1^)	N	%/Median (IQR)	N	%/Median (IQR)
Gender	Male	20	50%	10	50%	10	50%
	Female	20	50%	10	50%	10	50%
Smoking status	Never	22	55%	11	55%	11	55%
	Former	12	30%	6	30%	6	30%
	Current	6	15%	3	15%	3	15%
Education	Low	2	5%	0	0%	2	10%
	Medium	13	32.5%	9	45%	4	20%
	High	25	62.5%	11	55%	14	70%
Age (median, range)		40	60 (43; 77)	20	60 (44; 77)	20	60 (43; 76)
Body-mass index [kg/m^2^]		40	27.0 (22.9; 29.7)	20	26.5 (20.5; 27.8)	20	28.0 (24.0; 32.0)
Mini-Mental score (max = 30)				14	29.5 (28; 30)	–	–

1 Interquartile range.

Routine laboratory variables are listed in Table 2. The ALS cases demonstrated significantly higher levels of cholesterol, creatine kinase, gamma globulin, glutamate-oxaloacetate aminotransferase, and glutamate-pyruvate aminotransferase. The median concentrations of cholesterol and creatine kinase among the cases were higher than the cut-offs for the normal range. However, no differences in total immunoglobulins E, A, G, or M could be detected between cases and controls.

**Table 2:** Blood analysis of the study groups presented as the median and interquartile range (IQR).

	Standardvalue	Total				ALS patients				Non-diseasedcontrols				
		N	Median	IQR^1^		N	Median	IQR		N	Median	IQR		p value^2^
Alpha amylase [U/L]	<100	40	72	55.5	84.0	20	67.5	51.5	83	20	74	60.5	85.5	0.26
Albumin [g/L]	36–52	40	43.0	38.0	47.1	20	45.3	39.8	48.3	20	41.0	36.5	45.0	0.02
Alkaline phosphatase [U/L]	<129	40	78.0	68.0	87.0	20	76.0	62.0	84.5	20	81.5	74.5	91.0	0.16
Bilirubin [µmol/L]	<21	40	16.2	12.6	18.1	20	16.6	13.7	18.8	20	15.5	11.2	18.0	0.30
Calcium [mmol/L]	2.1–2.42	40	2.27	2.17	2.37	20	2.27	2.19	2.35	20	2.25	2.15	2.40	0.76
Cholesterol [mmol/L]	<5.2	40	5.23	4.77	5.67	20	5.62	5.23	5.88	20	4.82	4.43	5.20	<0.0001
Creatine kinase [U/L]	<175	40	145	114	180	20	180	151.5	272	20	122	90.5	140.5	<0.0001
Creatinine [µmol/L]	71–115	40	88.5	73.5	98.5	20	92.0	71.5	103.5	20	86.0	76.0	97.0	0.63
C-reactive protein [mg/L]	<5	40	3.20	2.25	4.00	20	3.50	2.85	4.30	20	2.95	2.15	3.60	0.14
Serum iron [µmol/L]	10.6–28.3	40	21.4	15.2	24.6	20	20.5	13.7	24.4	20	22.0	15.8	25.0	0.45
Gamma globulin [%]	6.2–15.4	40	12.8	10.2	14.4	20	14.1	12.4	14.5	20	11.5	9.8	13.1	0.01
Gamma-glutamyltranspeptidase [U/L]	<50	40	22.5	16.5	28.0	20	18.0	17.0	28.0	20	23.5	15.5	28.5	0.94
Blood sugar [mmol/L]	3.3–5.6	40	5.36	4.90	5.60	20	5.55	4.95	5.80	20	5.20	4.90	5.5	0.09
Glutamate-oxaloacetateaminotransferase [U/L]	<37	40	25.5	21.0	29.0	20	28.0	21.5	33.5	20	24.5	21.0	26.0	0.06
Glutamate-pyruvateaminotransferase [U/L]	<40	40	26	22.0	31.0	20	30.0	24.0	34.0	20	23.5	21.0	28.0	0.04
Hemoglobin [g/dL]	133–167	40	14.8	14.1	15.2	20	14.5	13.9	15.1	20	15.0	14.5	15.3	0.07
High-density cholesterol[mmol/L]	>1.0	40	1.28	1.15	1.46	20	1.22	1.11	1.47	20	1.35	1.23	1.5	0.19
Hematocrit [L/L]	0.39–0.55	40	0.48	0.44	0.51	20	0.45	0.42	0.51	20	0.49	0.45	0.51	0.22
Low-density cholesterol[mmol/L]	<3	40	3.03	2.88	3.3	20	3.18	2.87	3.45	20	2.99	2.88	3.1	0.30
Parathormone [pmol/L]	1.6–6.9	40	4.05	2.80	5.1	20	3.58	2.8	5.1	20	4.39	2.93	5.0	0.74
Triglycerides [mmol/L]	<1.7	40	1.64	1.45	1.89	20	1.75	1.42	2.21	20	1.6	1.45	1.69	0.12
Thyroid-stimulating hormone[mIU/L]	0.27–4.2	40	3.01	2.43	3.75	20	3.10	2.45	3.60	20	2.87	2.20	3.83	0.87
Carbamide nitrogen [mmol/L]	2.9–11.1	40	6.9	5.7	8.45	20	7.05	6	8.35	20	6.75	5.55	8.60	0.60
Uric acid [µmol/L]	200–416	40	315.5	245	346.5	20	326.5	270.5	373	20	284	229	331	0.12
White blood cell count [g/L]	3.7–9.5	40	7.65	6.4	8.8	20	7.23	6.05	8.7	20	7.82	6.74	8.95	0.19
Red blood cell sedimentationrate [mm/h]	1–12	40	8.0	5.5	10.0	20	8.0	5.5	10.0	20	8.0	5.5	10.5	0.98
Immunoglobulin E [IU/L]	<100	40	38.2	17.0	87.5	20	30.1	16.3	71.7	20	52.2	18.6	95.5	0.48
Immunoglobulin A [g/L]	0.85–4.5 (male),1.0–4.8 (female)	40	1.87	1.52	2.47	20	1.94	1.54	2.20	20	1.81	1.45	2.53	0.84
Immunoglobulin G [g/L]	8.0–17	40	9.52	8.51	10.85	20	9.27	8.51	10.54	20	9.83	8.84	11.32	0.22
Immunoglobulin M [g/L]	0.6–3.7 (male),0.5–3.2 (female)	40	1.05	0.77	1.39	20	1.08	0.75	1.69	20	0.97	0.78	1.27	0.47

1 Interquartile range,

2 Kruskal-Wallis test.

### Differences in the Immunoreactivity of IgG Antibodies

Differences in the immunoreactivity of IgG antibodies directed against human proteins were analyzed in the sera of 20 ALS patients relative to 20 controls. The serum from each individual in each group was probed using protein microarrays containing 9,480 different human proteins. Large numbers of immunoreactive antibodies were detected in both groups; therefore, and multistep statistical analysis was carried out in an attempt to identify high-level antibodies discriminating the study groups.

**Figure 1:**
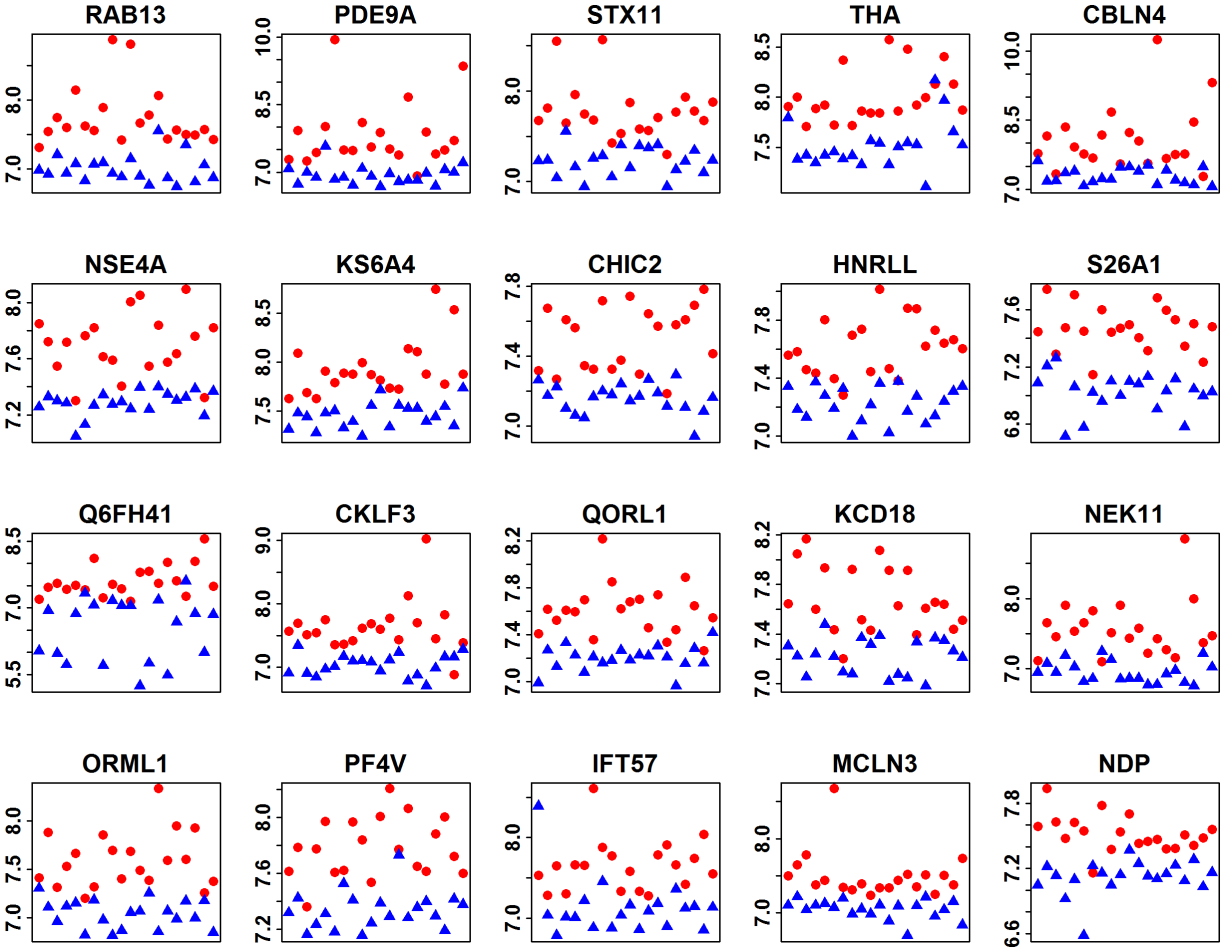
Fluorescence intensities of the antibodies bound to the selected 20 human proteins. Differential microarray fluorescence intensities (y-axes, log scale) of 20 antibodies bound to the selected proteins (identified by their respective gene names). ALS patient sera are shown in red (n = 20), and the control (NDC) sera are shown in blue (n = 20). The 40 samples (x-axes) were not paired. The samples were sorted by their sample labels only (ALS1, ALS2, …, ALS20, NDC1, NDC2, …, NDC20). Differences in these values indicate different reactivities of the IgG from the corresponding sera with the selected proteins.

We performed a statistical selection for discriminating the antibodies, as previously described. In each of the 100 subruns, 1 set of features was selected. The respective test set classification accuracy for these 100 feature panels ranged from 54.8% to 100%, with an average accuracy of 85.6%. Altogether, 207 features were selected at least once. The frequencies of the 20 most frequent protein features for all subruns are presented in Table 3. The most frequent protein was RAB 13, which was selected in 36% of all sub-runs. The next most frequent proteins were Syntaxin 11 and ribosomal protein S6 kinase, which were selected in 28% and 26% of the subruns, respectively. The least frequently selected protein was the BTB/POZ domain-containing protein, which was selected in 10% of all the subruns, the threshold set for the final potential biomarker candidates in this study. Among all 100 subruns, the most similar subrun panel comparing these 20 features contained 12 of these features and 19 additional proteins. For the corresponding subrun test set, the classification accuracy was 97.7%. The p values (see Table 3) of the 20 best-discriminating candidate proteins were <0.05 after adjustment for multiple testing using the method of Benjamini-Hochberg (FDR). The verification experiment to show that these 20 antibodies were discriminative as a panel for our microarray data set (100 RF classifications using 100 redrawn training and test set splits) yielded an average classification accuracy of 99.9% and an average sensitivity of 99.9%, and the corresponding specificities always proved to be 100%. Moreover, the fluorescence intensities of the 20 antibodies displayed a clear trend towards differentiating between ALS and control samples (Figure 1). As shown in Table 3, 14 of the 20 selected antibodies were also selected by PAM (i.e., position ≤245 of 9,480 in the ranked feature list obtained from PAM analysis), and 2 additional proteins were close to this selection level (positions 269 and 290).

**Table 3:** Proteins selected according to the high reactivity of IgG antibodies in the sera of ALS patients.

Database ID^1^	UniProt^1^	Gene name^1^	Protein feature description^1^	NDC^2^	ALS^3^	Freq^4^	PAM re-verification^5^	M Score p value^6^	M Score FDR^7^	M Score position^8^	t-test p value^9^	t-testFDR^10^
NM_002870.1	P51153	RAB13	RAB13, member RAS oncogene family	1028	1973	36%	86	1.1E-06	3.2E-03	2	4.8E-04	4.8E-03
NM_003764.2	O75558	STX11	Syntaxin 11	1383	2262	28%	56	5.0E-06	4.3E-03	9	5.0E-05	1.2E-03
NM_003942.1	O75676	KS6A4	Ribosomal protein S6 kinase alpha-4	1738	2642	26%	63	3.4E-05	8.6E-03	34	7.2E-05	1.5E-03
NM_144601.2	Q96MX0	CKLF3	CKLF-like MARVEL transmembrane domain containing 3 (CMTM3), transcript variant 1	1148	1970	25%	290	2.2E-04	1.6E-02	105	2.7E-03	1.6E-02
BC008217.1	Q8WVV9	HNRLL	Heterogeneous nuclear ribonucleoprotein L-like	1378	2023	21%	135	7.3E-05	1.0E-02	65	2.5E-07	5.3E-05
NM_134425.1	Q9H2B4	S26A1	Sulfate anion transporter 1	1140	1759	20%	95	1.2E-04	1.4E-02	72	3.1E-10	2.9E-06
NM_012110.1	Q9UKJ5	CHIC2	Cysteine-rich hydrophobic domain 2	1298	1934	19%	269	7.3E-05	1.0E-02	61	3.5E-07	6.6E-05
BC009047.1	O76083	PDE9A	Phosphodiesterase 9A	986	1872	16%	434	1.7E-06	3.2E-03	5	2.2E-02	7.3E-02
BC005212.1	Q9NXX6	NSE4A	Non-SMC element 4 homolog A	1475	2253	16%	74	2.3E-05	6.7E-03	28	7.1E-07	1.0E-04
NM_016467.1	Q9P0S3	ORML1	ORM1-like 1	1164	1929	14%	67	2.2E-04	1.6E-02	133	6.3E-06	3.6E-04
BC060765.1	Q8TDD5	MCLN3	Mucolipin 3	1191	1661	13%	512	1.6E-03	4.5E-02	325	2.0E-03	1.3E-02
NM_024800.1	Q8NG66	NEK11	Serine/threonine-protein kinase Nek11	1028	1796	12%	209	2.2E-04	1.6E-02	127	1.0E-03	8.1E-03
BC035137.1	P10827	THA	Thyroid hormone receptor, alpha	1768	2732	12%	43	9.7E-06	4.8E-03	18	1.2E-05	5.3E-04
NM_000266.1	Q00604	NDP	Norrie disease (pseudoglioma)	1258	1802	11%	158	4.2E-03	7.2E-02	542	2.4E-08	1.9E-05
NM_002620.1	P10720	PF4V	Platelet factor 4 variant 1	1501	2378	11%	38	3.9E-04	2.4E-02	149	1.9E-07	4.5E-05
BC013155.1	O95825	QORL1	Crystallin, zeta (quinone reductase)-like 1 (CRYZL1)	1353	2027	11%	147	2.2E-04	1.6E-02	107	3.1E-06	2.6E-04
NM_080617.4	Q9NTU7	CBLN4	Cerebellin-4	1368	2571	10%	517	2.0E-05	6.7E-03	25	3.2E-02	9.5E-02
NM_018010.2	Q9NWB7	IFT57	Intraflagellar transport 57 homolog	1167	2104	10%	500	5.3E-04	2.6E-02	155	1.9E-03	1.2E-02
NM_199334.2	Q6FH41/P10827	Q6FH41	Thyroid hormone receptor, alpha, transcript variant 1	953	1843	10%	19	1.8E-04	1.6E-02	101	5.2E-06	3.1E-04
BC059366.1	Q6PI47	KCD18	BTB/POZ domain-containing protein	1382	2069	10%	83	2.2E-04	1.6E-02	117	3.6E-06	2.8E-04

1 Obtained from Life Technologies.

2 Spot intensity median concerning control samples.

3 Spot intensity median concerning ALS samples.

4 Feature frequency within 100 feature selection subruns.

5 Position of the respective protein feature in the ranked feature list obtained from PAM analysis.

6 P-value obtained from an independently performed M score computation.

7 False discovery rate (method: Benjamini-Hochberg) for the M score p-values.

8 Feature position in the list of all ProtoArray features sorted by means of M score.

9 P-value obtained from an independently performed t test.

10 False discovery rate (method: Benjamini-Hochberg) for the t test p-values.

Overview of the 20 final selected proteins. The columns “Database ID” and “Description” show the corresponding specifications obtained from Life Technologies. The table is sorted by the means of feature frequency within 100 feature selection subruns (“Frequency”) used to select these reactive antibodies (cut-off: at least 10%). The columns “NDC” and “ALS” show the corresponding group-specific spot intensity medians, and the column ”PAM re-verification” gives the position of the respective protein feature in the ranked feature list obtained from PAM analysis. Additionally, the M scores (“M Score (p value)”) and t-test p values (“t test p value”) obtained from an independently performed 2-group comparison (i.e., not used for feature selection) using all samples (20 ALS vs. 20 NDC), the corresponding adjusted p values (“M Score FDR” and “t-test FDR”, method: Benjamini-Hochberg), and the corresponding positions of the features in the list of all ProtoArray features sorted by means of M score (“M Score Position”) are given for each feature to facilitate understanding of these p values.

### Clinical Data from the Patients with Lower and Higher Antibody Levels Directed Against the 20 Selected Proteins

The sera from 5 of the 20 ALS patients contained particularly high levels of immunoreactive IgG antibodies directed against the 20 selected proteins. Therefore, we compared the clinical data from this subgroup with the data from the remaining 15 ALS patients with lower reactivities (Table 4). However, the immunoreactivities of the sera from the 15 remaining patients were still higher than the reactivities of the sera from the control group.

**Table 4:** Data on the patients with lower and higher levels of detected potential biomarker antibodies.

	All ALS patients; N = 20	Low immunoreactivity; N = 15	High immunoreactivity; N = 5
Age (median, range)	59, 44–75	59, 44–75	52, 47–61
Female gender (N, %)	10, 50%	8, 53%	2, 40%
ALSFRS^1^ (median, range)	39, 16–46	40, 17–46	32, 16–40
Months between diagnosis andblood analysis (median, range)	15, 2–60	12, 2–60	24, 18–36
Bulbar signs (N, %)	11, 55%	7, 46%	4, 80%

1 Amyotrophic Lateral Sclerosis Functional Rating Scale.

The age of the ALS patients with lower levels of detected IgG antibodies was on average 59 years (median: 59, range 44–75) compared with 52 years for patients with higher immunoreactivities: (median: 52, range 47–61). The duration of the disease at the time of obtaining the blood samples in patients with lower immune reactivities was shorter (median: 12 months, range 2–60) compared to patients with higher immunoreactivities (median: 24 months, range 18–36). The ALSFRS for the patients with lower immune reactivities indicated a less severe disease stage (median: 40, range 17–46) than for patients with higher immune reactivities (median: 32, range 16–40). The typical disease symptoms and signs in the group with the higher immunoreactivities appeared earlier, and their disease was more severe with more functional deficits. The majority of the patients with higher immune reactivities demonstrated bulbar symptoms (4 out of 5), as compared 7 out of 15 of the patients with lower immune reactivities (Table 4).

## Discussion

### Inflammatory Mechanisms in ALS

The pathological hallmark of ALS is the degeneration of motor neurons in the spinal cord, brain stem, and motor cortex, although the cause of this degeneration currently remains unknown. Moreover, the brain is not accessible for the early detection of neurodegenerative diseases, and early clinical signs of motor function impairment may be associated with misclassification of this disease. Diagnostic serum markers are of special interest in supporting the early detection of this fatal disease. Here, we explored a large set of immunological serum markers to determine their performance in discriminating ALS patients from non-diseased subjects. We identified 20 candidate proteins that could differentiate between the study groups with 100% specificity and 99.9% sensitivity. Various biostatistical methods were developed and employed to control for overfitting [16], [22]–[24]. Notably, total immunoglobulin levels assessed using routine laboratory variables were not different between cases and controls.

Our results are in agreement with the current knowledge of the role of the immune system in ALS. A site-specific local inflammatory reaction was demonstrated in the regions of motor neuron degeneration both in ALS and in the SOD-1 transgenic mouse model [29], [30]. The main features of this inflammatory reaction were the accumulation of IgG in the cytoplasm of the motor neurons [29], the appearance of T cells [30] and antigen-presenting dendritic cells, an alteration in the cytokine profile in the affected tissue, and the activation of microglial cells and astrocytes [31]–[34]. The cellular and biochemical evidence of neuroinflammation in ALS patients and in animal models and the evidence of the role of neuroinflammation in initiating and amplifying the disease process and participating in the repair and protective processes were reviewed by Simpson *et al.* [33].

In the last 25 years, experimental autoimmune motor neuron diseases have been induced through the immunization of guinea pigs and goats with purified IgG from ALS patients [35]. When normal mice or guinea pigs are immunized with this IgG, the clinical ALS signs and similar ultrastructural alterations as those seen in humans with ALS can be observed [35].

In view of the wide spectrum of effects of anti-motor neuron IgG from ALS patients in animal models, a set of antibodies may hold promise as a biomarker panel for the early detection of ALS and possibly for monitoring disease progression. Our approach made use of the advantage of a high-density protein microarray with extensive bioinformatic analysis to search for a set of high-level antibodies as markers differentiating 20 ALS patients from 20 healthy subjects. The details of our bioinformatics tools have been published elsewhere [17], [24]–[26].

### Critical Points - Study Design and Antibodies as Potential Biomarkers

To the best of our knowledge, this was the first study to evaluate the humoral immune response in the form of IgG autoantibodies for ALS profiling. In our study, the patient groups each consisted of 20 subjects, with the controls matched to the cases based on age and gender. Matching by age is important because the number of antibodies directed against self-antigens increases with age. Subjects taking immune-modulating drugs were excluded, and the serum preparations for cases and controls were performed in parallel and in a blinded manner according to a standardized protocol. This approach was critical to avoid batch problems and other forms of bias in the detection of disease-related antibodies [27], [28].

### The Selection of Potential Biomarker Candidates

Our results demonstrated that the 20 ALS patients included in this study could be distinguished from the controls through the use of a small panel of 20 highly immunoreactive IgGs directed against 20 selected human proteins. The approach of repeated resampling and the frequency of the selections led to the acquisition of the most reproducible set of candidate markers for our dataset. The robustness of these markers to serve as diagnostic markers was re-verified independently using an alternative feature selection strategy involving the use of PAM. Finally, the average classification accuracy of 99.9% (based on 100 repeatedly resampled test/training sets and the 20 markers) verified that the panel was highly discriminatory for our data set. Nevertheless, our results should be regarded as preliminary, and studies with more ALS patients and controls and prospective studies with asymptomatic subjects at baseline are needed to validate these candidate biomarkers. Although we excluded patients taking immunotherapies, many ALS patients receive different medications and have elevated liver enzymes. Moreover, cross-sectional studies have methodological shortcomings for biomarker research [36], and prospective studies require international networks to gain sufficient power in biomarker studies for the early detection of rare diseases like ALS.

### Levels of Antibodies in ALS Patients

The high level of antibodies directed against the 20 selected human proteins appeared to be characteristic of the group of our ALS patients, as these antibodies were not found to be elevated in the age- and gender-matched controls. This antibody production may be a consequence of the immune response to proteins released from dying motor neurons. Because ALS involves a long preclinical phase in which motor neurons are subject to decay, the appearance of high-level antibodies directed against the 20 selected human proteins may predict the development of the disease. Indeed, patients with higher antibody levels presented with more severe symptoms as compared to patients with lower antibody levels. The patients with high antibody levels were also more prone to exhibit bulbar symptoms. This finding may be due to a longer time between diagnosis and the measurement of the antibody levels, which was on average 12 months later than in patients with lower antibody levels. These findings may suggest that increasing autoantibody levels could be used to track the progression of ALS disease, and future longitudinal studies in ALS patients could confirm this observation.

### The Potential Role of Antibodies in ALS

*It should be noted that we have no clear-cut evidence that any or all of the 20 identified autoantibodies are pathogenic in ALS. Nevertheless, there are several examples in which pathogenic antibodies are generated alongside nonpathogenic antibodies in patients with different diseases. The best such example is systemic lupus erythematosus, although multiple pathogenic antibodies can also be a feature of myasthenia gravis, in which antibodies directed against different antigens of the neuromuscular junction are found simultaneously with antibodies directed against different antigens of the thyroid glands or mucous membrane of the stomach. The 20 identified proteins in our study have nothing in common with known proteins involved in ALS inflammation; however, the proteins identified in the present study are mostly intracellular, intra-motor neuron proteins, indicating that the target of the humoral immune response may be the motor neuron itself.*

We could not confirm previous reports that identified antibodies with alternative molecular targets such as neurofilaments, Fas (CD95), fetal muscular proteins, vascular antigens, matrix metalloproteinases, and calcium channel proteins, as reviewed by Pagani *et al.,* with the exception of mucolipin3 [6]. Nevertheless, it is noteworthy that the intracellular locations of the proteins selected in this study are largely confined to those subcellular organelles to which IgG binds intracellularly in ALS motor neurons [34] and in mice injected with IgG from ALS patients [5]. Furthermore, such inoculated mice exhibited electrophysiological and morphological alterations that were similar to those observed in ALS patients.

### The Sites and Normal Functions of the Selected Proteins

**RAB 13** regulates endocytosis, membrane trafficking between the trans-Golgi network and recycling endosomes, and neurite outgrowth and regeneration [37], [38]. Early endosomal membrane compartments are required for the formation and recycling of synaptic vesicles, and fragmentation of the Golgi apparatus is an early event in the pathogenesis of neuronal degeneration in ALS. However, the nature of the involvement of the Golgi in the pathogenic mechanism of neurodegenerative disease remains unknown [39]. Furthermore, the following 3 selected proteins are also localized to the Golgi network.

**Syntaxin 11** is associated with late endosomes and the trans-Golgi network [40] and is localized to synaptic vesicles [41]. When IgG from ALS patients is administered intraperitoneally to mice, the IgG is taken up in the motor axon terminals, binds to synaptic vesicles, increases the intra-terminal level of Ca^(2+)^, and accumulates in the Golgi, which is dilated and displays an elevated Ca^(2+)^ content [5], [35].

The **cysteine-rich hydrophobic domain 2** protein is also localized to the trans-Golgi network [42]. In fact, the Golgi network is injured in one of the familial forms of ALS. Optineurin, which is mutated in ALS 12, is responsible for maintaining the integrity of the Golgi network, and this protein also plays a role in exocytosis by interacting with RAB proteins [43]. Moreover, mutation of the Alsin gene in familial ALS 2 impairs endocytosis [44].

The **intraflagellar transport 57 homolog** protein is part of the motor for retrograde axonal transport, and abnormalities in this system have been linked to ALS. Defects in dynein-mediated axonal transport have also been shown in transgenic SOD1 mice, which are one of the models of genetic ALS [45]. Dynein is the motor for the movement along microtubules, and different mutations in dynactin, which attaches microtubules to dynein, increase susceptibility to ALS [46]. IgG has also been detected bound to microtubules both in spinal motor neurons in SALS and also in mice inoculated with IgG from ALS patients [5]. However, whether this phenomenon is partially responsible for the slowed retrograde axonal transport in motor neurons in SALS is currently unknown.

The rough endoplasmic reticulum (RER) is another subcellular structure that is heavily loaded with IgG in motor neurons in human ALS patients and animal models [5], [34]. IgG appears to be bound both to the ribosomes and to the membranes of cisternae of the RER. Furthermore, the RER is vacuolar, and its Ca^(2+)^ content is increased [35].

One of the selected proteins in the RER is the **ribosomal protein S6 kinase**. This protein takes part in signal transduction and is a substrate of a ubiquitous and versatile mediator of extracellular signal-regulated kinase, which is activated by growth factors, peptide hormones, and neurotransmitters [47].

Another selected protein localized to the RER is the **ORM1-like 1** protein, which may indirectly regulate endoplasmic reticulum-mediated Ca^(2+)^ signaling [48], [49].

Structural alterations and dysfunction of the above-mentioned intracellular structures have recently been discovered in distinct genetic forms of ALS. If intracellular antibodies are bound to other proteins in these structures, the functions of these structures may also be altered.

**Mucolipin 3** belongs in a family of ion channel proteins that are expressed in endosomes and lysosomes. This protein is a novel Ca^(2+)^-permeable channel that releases Ca^(2+)^ from endosomes and lysosomes. This protein additionally plays a role in the regulation of cargo trafficking along the endosomal pathway [50]. When IgG from the sera of ALS patients was injected in mice, this IgG bound to and accumulated in the lysosomes and endosomes of the motor neurons [5].

**Cerebellin** family members are known to act as transneuronal regulators of synapse development and synaptic plasticity in various brain regions. Decreased concentrations of cerebellin have been observed in the brains of patients with olivopontocerebellar atrophy and Shy-Drager syndrome, suggesting a role for cerebellin in the pathology of these diseases [51]. In certain ALS patients, ALS plus syndrome develops in the late stage of the disease and includes certain signs of Parkinson’s disease. Neuropathological data indicate that the cerebellum may also be involved in the neurodegenerative process in ALS, although the symptoms and signs of the severe motor neuron degeneration may conceal the cerebellar dysfunction [52].

**Serine/threonine-protein kinase NEK 11** is involved in the genotoxic stress response and DNA repair [53]. **None-SMC element 4 homolog A,** (the non-structural maintenance of chromosomes element 4 homolog A) similarly takes part in DNA repair [54].

Missense mutation of the **alpha** **crystallin** domain in the small heat shock protein HSP22 causes motor neuron-specific neurite degeneration and alpha-B crystallin accumulation in ballooned neurons in neurodegenerative diseases [55], [56].

**Zeta-crystallin**, a quinone oxidoreductase, is related to Torpedo and the mammalian synaptic vesicle membrane protein VAT-1. VAT-1 is a major protein of the synaptic vesicles from Torpedo [57]. IgG from the sera of ALS patients is taken up in the axon terminal of the motor neurons and binds to the synaptic vesicles [5].

**Platelet factor 4 variant 1** is a potent regulator of endothelial cell biology that affects angiogenesis and vascular diseases [58]. A loss-of-function mutation in the angiogenin gene, another regulator of angiogenesis, has been described in ALS patients [59]. The alterations to the blood-brain barrier in ALS were recently extensively reviewed [60]. ALS IgG administered into mice was observed by electron microscopy and immunohistochemistry to be bound to the surface of the endothelial cells and transported in multivesicular bodies inside of the cells [5].

**The BTB/POZ domain-containing protein** is a protein containing a common structural domain. Proteins that interact with the BTB/POZ domain inhibit the death of motor neurons in SOD mutant transgenic mice [61].

**Thyroid hormone receptor alpha** is a nuclear protein that binds triiodothyronine. Autoimmune thyroid diseases have been found to complicate ALS [62].

## Conclusions

A panel of highly expressed IgG antibodies obtained from serum samples was found to differentiate ALS patients from controls with high specificity and sensitivity, and increasing disease severity was associated with higher antibody levels in ALS cases. Therefore, this panel may be of value as a potential biomarker to use in diagnostics and in the monitoring the ALS disease progression. However, validation of the prognostic features of these candidate biomarkers in a prospective study with ALS patients is important. Prospective studies on the diagnostic features of these biomarkers should take into account the rare nature of ALS, which results in low positive predictive values for diagnostic tests. The application of a fluidic microarray platform is also an important issue for future research.

### Data Availability

The data are available at www.medizinisches-proteom-center.de/May_et_al.

## Supporting Information

Figure S1 Data analysis workflow. The main data analysis workflow is outlined (without re-verification using PAM). The automatic biomarker selection (“feature selection”, dashed box on the left) was composed from 100 iterative subruns. In each subrun, the following 5 steps were performed: (1) random drawing of the new test (6 ALS and 6 NDC samples) and new training sets (14 ALS and 14 NDC samples); (2) preselection of the 300 best features with regard to M Scores from this training set (corresponding average M Score cut-off: 0.004566); (3) further narrowing by gene shaving (GS) using only the training set drawn in (1); (4) use of the resulting features (“subrun selection”) to train a random forest classifier (RF, using only the training set drawn in (1); and (5) use of this classifier to predict the test set drawn in step (1) to verify the subrun selection. These 100 subruns resulted in 100 distinct feature sets (i.e., subrun selections) selected from their subrun-specific training set consisting of 1 to 45 proteins and verified by prediction of their subrun-specific test set (average accuracy: 85.6%). The occurrence of each feature in such a subrun selection was next counted for a feature ranking concerning the overall selection frequency. All subrun-selected features with a frequency of at least 10% were reported in the final set of proteins (20 features). Finally, these features were verified by RF training and classification (test and training set redrawn from all samples, 100 times repeated) with an average accuracy of 99.9% and re-verified by PAM analysis (not shown in this Figure).
(TIF)

Figure S2 Technical replicates. Quantile plots (log data) of the worst (r = 0.9688) and best (r = 0.9914) pairs out of 12 pairs of technical replicates analyzed in a preliminary study are shown.
(TIFF)

Figure S3 Heat map of the immunoreactivities of the antibodies from the sera of ALS patients and NDCs. This heat map shows the microarray fluorescence intensities (log scale) of the IgGs from the 20 ALS patients and 20 non-diseased controls bound to the selected 20 proteins (identified by their respective gene names). The ALS sera (n = 20, on the right) and non-diseased control sera (n = 20, on the left) are shown in heat color representation (red = low values, white/yellow = high values). The fluorescence values of all antibodies were higher in total in the ALS group compared to the non-diseased control group, suggesting higher immune reactivity.
(TIFF)

File S1 Supporting information. Table S1, Results of a technical replicates study. The results of a preliminary study with technical replicates (8 microarrays, two different serum samples, two different ProtoArray production lots) are shown. For all pairs of technical replicates Pearson’s correlation coefficient (for log data) and the average coefficient of variation (CV, for raw data) have been computed. Due to batch effects the intra-lot reproducibility (r = 0.9883, average CV = 9.01%) is better than the inter-lot reproducibility (r = 0.9729, average CV = 11.61%) and the overall reproducibility (r = 0.9780, CV = 10.74%).
(DOCX)
