# Supplementary figures and images for: Highly Immunoreactive IgG Antibodies Directed against a Set of Twenty Human Proteins in the Sera of Patients with Amyotrophic Lateral Sclerosis Identified by Protein Array
Source: PLoS One. 2014 Feb 26;9(2):e89596. doi: 10.1371/journal.pone.0089596 (PMC3935926; doi:10.1371/journal.pone.0089596)

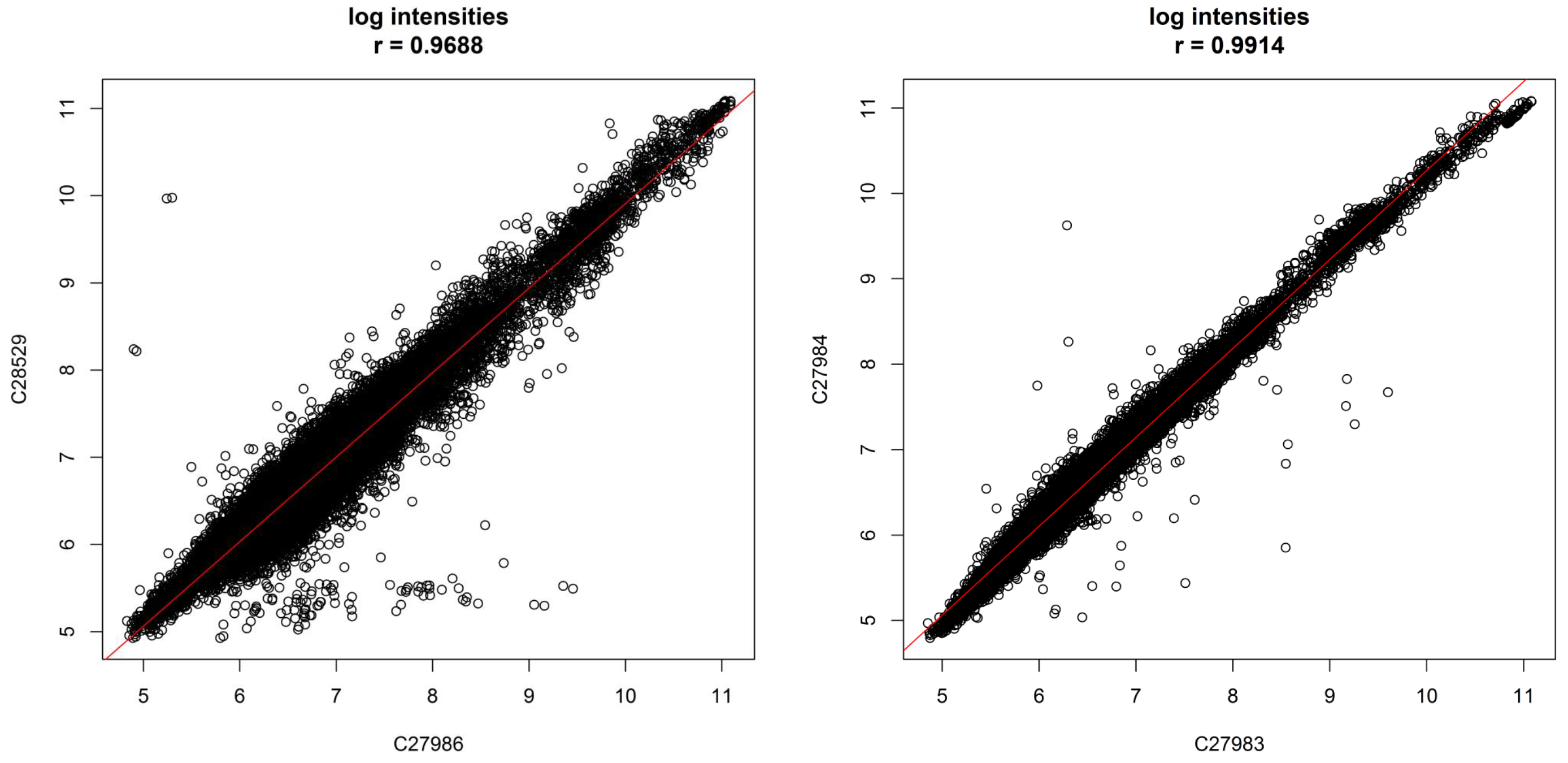

Supplement: Figure S2 — Technical replicates. Quantile plots (log data) of the worst (r = 0.9688) and best (r = 0.9914) pairs out of 12 pairs of technical replicates analyzed in a preliminary study are shown. (TIFF) [file pone.0089596.s002.tiff]

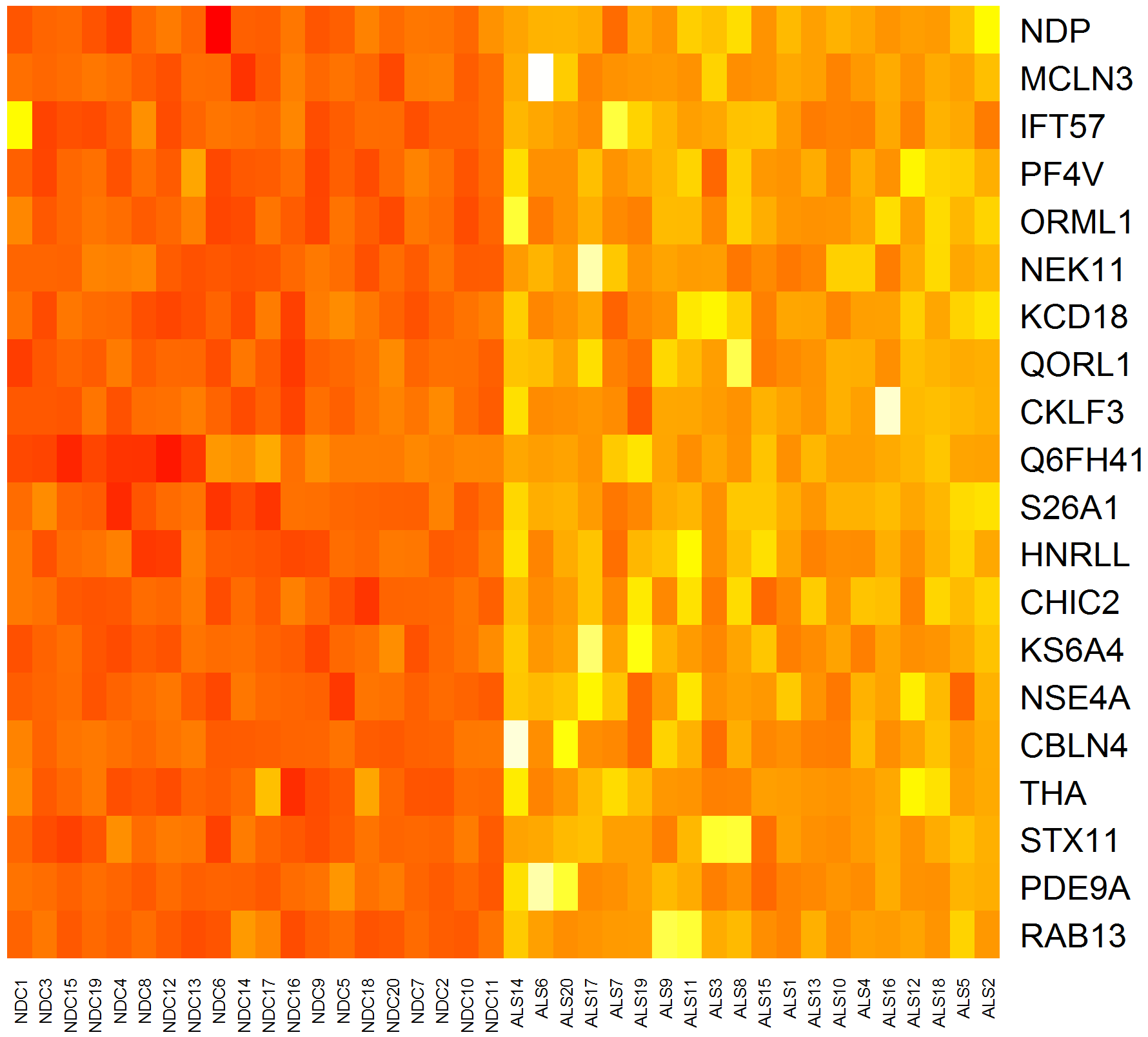

Supplement: Figure S3 — Heat map of the immunoreactivities of the antibodies from the sera of ALS patients and NDCs. This heat map shows the microarray fluorescence intensities (log scale) of the IgGs from the 20 ALS patients and 20 non-diseased controls bound to the selected 20 proteins (identified by their respective gene names). The ALS sera (n = 20, on the right) and non-diseased control sera (n = 20, on the left) are shown in heat color representation (red = low values, white/yellow = high values). The fluorescence values of all antibodies were higher in total in the ALS group compared to the non-diseased control group, suggesting higher immune reactivity. (TIFF) [file pone.0089596.s003.tiff]
