# Supplementary material for: Highly Immunoreactive IgG Antibodies Directed against a Set of Twenty Human Proteins in the Sera of Patients with Amyotrophic Lateral Sclerosis Identified by Protein Array
Source: PLoS One. 2014 Feb 26;9(2):e89596. doi: 10.1371/journal.pone.0089596 (PMC3935926; doi:10.1371/journal.pone.0089596)
Supplement: File S1 — Supporting information. Table S1, Results of a technical replicates study. The results of a preliminary study with technical replicates (8 microarrays, two different serum samples, two different ProtoArray production lots) are shown. For all pairs of technical replicates Pearson’s correlation coefficient (for log data) and the average coefficient of variation (CV, for raw data) have been computed. Due to batch effects the intra-lot reproducibility (r = 0.9883, average CV = 9.01%) is better than the inter-lot reproducibility (r = 0.9729, average CV = 11.61%) and the overall reproducibility (r = 0.9780, CV = 10.74%). (DOCX) [file pone.0089596.s004.docx]

# Supplement

## This supplement describes a preliminary technical replicates study and the biomarker candidate identification workflow in detail. Figure S1 shows the data analysis workflow.

## Preliminary technical replicates study

## To check the reproducibility of the microarrays, technical replicates were used. In a preliminary study, 2 different serum samples were analyzed with 4 different arrays each (2 from ProtoArray production lot 1 and 2 from a lot 2). Hence, altogether 8 microarrays were incubated and 12 different pairs of technical replicates were obtained. For each pair of technical replicates, the Pearson correlation coefficient (using log data) and the mean coefficient of variation (using raw data) were computed (see Table S1). Additionally, quantile plots were drawn (see Figure S2). The results of this preliminary study showed better intra-lot reproducibility (r = 0.9883, average CV = 9.01%) than inter-lot reproducibility (r = 0.9729, average CV = 11.61%). We therefore decided to use microarrays from one ProtoArray lot only. Finally, an overall average correlation coefficient of r = 0.9780 and an overall average coefficient of variation of CV = 10.74% were obtained. These results demonstrate good reproducibility for our microarrays.

## Methods for biomarker candidate selection

**Gene shaving:** The 300 proteins resulting from the univariate preselection (see main manuscript) were narrowed down by multivariate selection, using a random forest (RF) classifier wrapped with a backward elimination approach (“gene shaving” (GS), [6-8]). The method used in this study is an adaption of the approach proposed by Jiang. *et al.* [8]. GS starts with the set of all preselected protein features $S_{1}$. In the following iterations, RF models are trained and evaluated (10-fold cross-validation) on variable set $S_{i}$ to obtain the variable ranking vector ${importance}_{i}$ and the classification accuracy ${accuracy}_{i}$ for that variable set. The 10% weakest variables are then discarded to obtain $S_{i+1}$. Finally, the variable set ($S_{best}$) with the best accuracy value (${accuracy}_{best}$) is obtained and classification is performed within the independent test set for reporting the overall accuracy. Hence, the selected proteins were verified by classification. For this GS approach, the R package *randomForest* [9] in combination with own R code was used. To ensure that the RF results were as stable as possible, the number of trees in the forest (parameter *ntree*) was set to 3p (where p is the current number of predictors) and the number of predictors sampled for splitting at each node (parameter *mtry*) was set to p/3. In consequence of the biological variance of the antibodies, undersampling (“n<<p”), overfitting and correlated variables, the feature selection and verification results varied with different test and training set splits. This instability is a general issue of microarray studies [10,11]. To minimize this bias and select more reliable biomarker candidates, the whole feature selection and verification procedure (including the test and training set split and the preselection) was repeated 100 times (100 “subruns”). The final biomarker candidate set was assembled from the features that were most frequent in the 100 subrun selections (“frequency of selections”-based approach, [11]). 100 new test and training sets were then redrawn randomly. 100 RF models were built from the final biomarker candidate set on the 100 training sets and were used to determine the accuracy on the respective 100 test sets to yield the verification that these biomarker candidates were discriminative as a panel for our data.

**PAM analysis**: Additionally, the obtained set of biomarker candidates was reverified by an alternative data analysis approach. As in other recent ProtoArray studies [12,13], the Prediction Analysis of Microarrays (PAM, <http://www-stat.stanford.edu/~tibs/PAM/>) approach implemented in the R package pamr (<http://cran.r-project.org/web/packages/pamr/index.html>) was used as a reverification method. PAM uses the approach of nearest shrunken centroids [14] for feature selection and classification. In this study the following PAM analysis was performed: After training a PAM model with default settings using all samples and all 9480 protein features, a convenient threshold was determined by using the function pamr.cv which performs a cross-validated parameter optimization. All features satisfying this threshold were then regarded as discriminative predictors for ALS vs. NDC. Among these features, those that were also selected by GS were determined to reverify these biomarker candidates.

## Results of biomarker candidate selection

In consequence of the different test and train set splits, different sets containing the respective 300 best features according to the M statistic were obtained during the preselection step of each feature selection subrun. Accordingly, altogether 1616 of 9480 features were preselected at least once, with an average frequency of occurrence of 18.56. On the other hand, 201 features were selected at least 50 times, and only 17 features occurred in all 100 preselected sets.

During each subrun 47 feature subsets $S_{i}$ were evaluated by GS until $S_{47}$ consisted of only one feature. The cardinality of the 100 reported $S_{best}$ sets ranged from 1 up to 45 features, and their corresponding classification accuracy (obtained from 10-fold cross-validation) was always 100%. Furthermore, their corresponding test set classification accuracy ranged from 54.75% to 100%, with an average accuracy of 85.58% (standard deviation: 10.69). Altogether 207 features were selected at least once with an average frequency of 3.55%. Hence, analogously to the preselection, most of them (here: 165) showed low frequencies (i.e. were selected less than 5 times). Moreover, no feature with a frequency of more than 50% was reported. The frequencies of the 20 most frequent protein features in all reported $S_{best}$ are shown in Table 3. The most frequent protein was RAB13, which was reported in 36% of all the $S_{best}$ sets. The two next most frequent proteins were obtained in 28% and 26% of the subruns, respectively. The least frequent feature is KCTD18, which was reported in 10% of all the $S_{best}$ sets, which was the threshold for the final biomarker candidate set in this study. The verification experiment that these 20 biomarker candidates were discriminative as a panel for our data (100 random forest classifications using 100 redrawn train and test set splits) yielded classification accuracies ranging from 99.25% to 100%, with an average accuracy of 99.9% (standard deviation: 0.00075). The corresponding sensitivities ranged from 98.5% to 100%, with an average sensitivity of 99.9%, and the corresponding specificities were always 100%. Moreover, the fluorescence intensities of the 20 biomarker candidates showed a clear tendency to being differential for the ALS and NDC samples (see Figure 1 and Figure S3).

Finally, the results of the PAM reverification analysis showed that most of the 20 selected potential biomarker candidates were discriminative for ALS and NDC. After PAM model training the threshold 2.394 was identified as convenient, because it provided a good tradeoff between classification error and feature set cardinality (about 10% classification error and 245 features) for our data set. As may be seen in Table 3, 14 of the 20 selected potential biomarker candidates were also selected by PAM (i.e. position ≤ 245 of 9480 in the ranked feature list obtained from PAM analysis). Two more features were close to that selection (positions 269 and 290).

## Discussion of biomarker candidate selection

The preselected feature sets obtained during the 100 feature selection subruns proved to be unstable and highly dependent on the different training samples. Consequently, obvious instability of the overall feature selection was observed. However, the stability of feature selection and classification accuracy results is a known issue in microarray data analysis [10,11] and machine learning applications. Nevertheless, it was ignored in recent protein microarray studies and biomarker selections based on a single split in training and test set were published (e.g. in [12,13]). As a consequence, the results of these studies are not comparable with our findings. To overcome the instability problem, Michiels *et al.* [10] propose the use of verification by repeated resampling, and Baek *et al.* [11] recommend the combination of resampling with a frequency of selections approach Accordingly, the most frequently selected features should be the most relevant biomarker candidates. We have adopted these recommendations in our feature selection approach so as to obtain reliable biomarker candidates.

The results of our biomarker identification demonstrated that for our data set the ALS patients could be distinguished from the NDCs through the use of a small amount of serum and a small panel of 20 biomarker candidates. The average classification accuracy of 85.6% based on the 100 feature selection subruns is slightly higher than respective accuracies reported by Baek *et al.* [11] for RF-based feature selection (75.06%-85.48%, also 100 subruns). On the other hand, their selected feature panels are more stable (a standard deviation of 2.46-9.38 vs. 10.69 in this study). These differences may be caused, for example, by differences in microarray technology (oligonucleotide arrays vs. protein arrays), sample sizes (n=62 vs. n=40), verification strategy (100 cross-validation procedures vs. 100 split-sample procedures), target diseases (colon cancer vs. ALS) and biological variation. However, as a result of our repeated resampling and frequency of selection approach we ensured the isolation of the most stable and biologically most relevant set of 20 biomarker candidates. The relevance of most of these biomarker candidates was reverified independently by an alternative feature selection strategy using PAM. Finally, the average classification accuracy of 99.9% (standard deviation: 0.00075, sensitivity: 99.9%, specificity: 100%) based on 100 repeatedly resampled test and training sets (from all 20 ALS vs. 20 NDC arrays) and the 20 potential biomarker candidates verified that the panel was discriminative for our data set. Nevertheless, a validation study involving the use of completely different samples is needed to validate these biomarker candidates.

References

1. R Development Core Team "Team RDC" (2011) R: A Language and Environment for Statistical Computing. Vienna, Austria: R Foundation for Statistical Computing.

2. Gentleman RC, Carey VJ, Bates DM, Bolstad B, Dettling M, et al. (2004) Bioconductor: open software development for computational biology and bioinformatics. Genome Biol 5: R80.

3. Smyth GK (2005) Limma: linear models for microarray data. In: Gentleman R, Carey V, Dudoit S, Irizarry R, Huber W, editors. Bioinformatics and Computational Biology Solutions using R and Bioconductor. New York: Springer. pp. 397-420.

4. Sboner A, Karpikov A, Chen G, Smith M, Mattoon D, et al. (2009) Robust-linear-model normalization to reduce technical variability in functional protein microarrays. J Proteome Res 8: 5451-5464.

5. Love B (2007) The Analysis of Protein Arrays. Functional Protein Microarrays in Drug Discovery: CRC Press. pp. 381-402.

6. Díaz-Uriarte R, Alvarez de Andrés S (2006) Gene selection and classification of microarray data using random forest. BMC Bioinformatics 7: 3.

7. Hastie T, Tibshirani R, Eisen MB, Alizadeh A, Levy R, et al. (2000) 'Gene shaving' as a method for identifying distinct sets of genes with similar expression patterns. Genome Biol 1: RESEARCH0003.

8. Jiang H, Deng Y, Chen HS, Tao L, Sha Q, et al. (2004) Joint analysis of two microarray gene-expression data sets to select lung adenocarcinoma marker genes. BMC Bioinformatics 5: 81.

9. Breiman L (2001) Random forests. Machine Learning 45: 5-32.

10. Michiels S, Koscielny S, Hill C (2005) Prediction of cancer outcome with microarrays: a multiple random validation strategy. Lancet 365: 488-492.

11. Baek S, Tsai CA, Chen JJ (2009) Development of biomarker classifiers from high-dimensional data. Brief Bioinform 10: 537-546.

12. Nagele E, Han M, Demarshall C, Belinka B, Nagele R (2011) Diagnosis of Alzheimer's disease based on disease-specific autoantibody profiles in human sera. PLoS One 6: e23112.

13. Han M, Nagele E, Demarshall C, Acharya N, Nagele R (2012) Diagnosis of Parkinson's disease based on disease-specific autoantibody profiles in human sera. PLoS One 7: e32383.

14. Tibshirani R, Hastie T, Narasimhan B, Chu G (2002) Diagnosis of multiple cancer types by shrunken centroids of gene expression. Proc Natl Acad Sci U S A 99: 6567-6572.

**Tables**

**Table S1 Results of a technical replicates study**

| **Array 1** | **Array 2** | **Lot array 1** | **Lot array 2** | **Serum** | **Mean CV** | **Pearson’s r** |
| --- | --- | --- | --- | --- | --- | --- |
| C27985 | C28527 | HA20238 | HA20240 | serum1 | 12.22% | 0.9701 |
| C27985 | C28529 | HA20238 | HA20240 | serum1 | 10.71% | 0.9749 |
| C27986 | C28527 | HA20238 | HA20240 | serum1 | 11.73% | 0.9703 |
| C27986 | C28529 | HA20238 | HA20240 | serum1 | 12.50% | 0.9688 |
| C27983 | C28530 | HA20238 | HA20240 | serum2 | 13.80% | 0.9735 |
| C27983 | C28531 | HA20238 | HA20240 | serum2 | 11.71% | 0.9753 |
| C27984 | C28530 | HA20238 | HA20240 | serum2 | 10.13% | 0.9750 |
| C27984 | C28531 | HA20238 | HA20240 | serum2 | 10.09% | 0.9750 |
| C27986 | C27985 | HA20238 | HA20238 | serum1 | 7.86% | 0.9894 |
| C28527 | C28529 | HA20240 | HA20240 | serum1 | 9.96% | 0.9835 |
| C27984 | C27983 | HA20238 | HA20238 | serum2 | 10.04% | 0.9914 |
| C28530 | C28531 | HA20240 | HA20240 | serum2 | 8.17% | 0.9890 |

The results of a preliminary study with technical replicates (8 microarrays, two different serum samples, two different ProtoArray production lots) are shown. For all pairs of technical replicates Pearson’s correlation coefficient (for log data) and the average coefficient of variation (CV, for raw data) have been computed. Due to batch effects the intra-lot reproducibility (r = 0.9883, average CV = 9.01%) is better than the inter-lot reproducibility (r = 0.9729, average CV = 11.61%) and the overall reproducibility (r = 0.9780, CV = 10.74%).
